# Surface Functionalization by Hydrophobin-EPSPS Fusion Protein Allows for the Fast and Simple Detection of Glyphosate

**DOI:** 10.3390/bios9030104

**Published:** 2019-08-29

**Authors:** Julia Döring, David Rettke, Gerhard Rödel, Tilo Pompe, Kai Ostermann

**Affiliations:** 1 Institute of Genetics, Technische Universität Dresden, Zellescher Weg 20b, 01217 Dresden, Germany; 2 Institute of Biochemistry, Leipzig University, Johannisallee 21-23, 04103 Leipzig, Germany

**Keywords:** glyphosate, malachite green assay, hydrophobin, EPSPS, immobilization

## Abstract

Glyphosate, the most widely used pesticide worldwide, is under debate due to its potentially cancerogenic effects and harmful influence on biodiversity and environment. Therefore, the detection of glyphosate in water, food or environmental probes is of high interest. Currently detection of glyphosate usually requires specialized, costly instruments, is labor intensive and time consuming. Here we present a fast and simple method to detect glyphosate in the nanomolar range based on the surface immobilization of glyphosate’s target enzyme 5-enolpyruvylshikimate-3-phosphate synthase (EPSPS) via fusion to the hydrophobin Ccg2 and determination of enzyme activity with a malachite green assay, which is a common photometric technique to measure inorganic phosphate (Pi). The assay demonstrates a new approach for a fast and simple detection of pesticides.

## 1. Introduction

Glyphosate is a potent post-emergent total herbicide. It belongs to the group of organophosphonate-pesticides and is one of the most widely used herbicides worldwide. There is an ongoing discussion about its impact on human health [1,2,3] and on environment [4,5,6]. In 2014, an accredited but not peer-reviewed study was published reporting on the detection of glyphosate in breast milk of American mothers [7]. Glyphosate was also found in beer [8] and urine samples [9,10]. However, these findings could not be confirmed by some other studies [11,12,13]. Furthermore, there are studies showing an effect of glyphosate or its formulations for example on human placental cells and aromatase [14] or on cell adhesion properties [15]. Different results in several studies on glyphosate and its assessment of the International Agency for Research on Cancer (IARC) [16] as a probably carcinogenic compound raised the debate about the hazardous risks and the approval of glyphosate in the European Union. Glyphosate detection is therefore of great importance to reveal detailed information about its distribution. Because of its physicochemical properties, i.e., its small size, its polarity and the high water solubility, the detection of glyphosate is difficult. Furthermore, it is non-volatile and zwitterionic [17,18]. Most available methods for detection of glyphosate are costly in terms of sample preparation, technical equipment and time consumption. Moreover, they require qualified personnel as detection mostly relies on ELISA techniques or chromatography methods coupled with mass spectrometry [10,19,20,21].

Some approaches have been developed to detect glyphosate using colorimetric techniques. In general, they are based on the reaction of glyphosate with carbon dioxide or another substrate to form a dithiocarbamic acid intermediate which can be detected by different read out systems [22,23,24].

None of these techniques exploits the specific target of glyphosate, the 5-enolpyruvylshikimate-3-phosphate synthase (EPSPS) [25,26,27]. This enzyme is part of the shikimate pathway, which is present in plants and some microorganisms and is responsible for the formation of aromatic amino acids [28]. It uses shikimate-3-phosphate (S3P) and phosphoenolpyruvate (PEP) as substrates to produce 5-enolpyruvylshikimate-3-phosphate (EPSP) and inorganic phosphate (Pi) [29]. Several studies suggest that glyphosate occupies the binding site for PEP keeping the enzyme inactive in an EPSPS-S3P-glyphosate intermediate ternary complex [30,31,32].

As described above, EPSPS enzymatic activity is accompanied by the formation of Pi. An option to detect and quantify Pi in the nanomolar range is the malachite green assay [33,34]. Therefore it might be suited to measure the enzymatic activity of EPSPS and its inhibition by glyphosate. Compared to other assays for Pi quantification, e.g., the molybdenum blue method, the malachite green assay is easier to handle and exhibits a higher sensitivity [33,35]. The assay is based on the formation of a complex of phosphomolybdate malachite green, which has a greenish blue color and absorbs in the range between 620 to 660 nm [33]. Baykov et al. [36] were able to simplify the procedure by increasing the amount of sulfuric acid in the malachite green solution so that filtration of the dye solution is no longer necessary.

To develop a fast, easy to handle, robust and compact detection assay, it might be advantageous to immobilize the detection unit (here: EPSPS) to create a ready-to-use chip. Furthermore, protein immobilization often can increase protein stability [37] and improve substrate accessibility [38]. On the other hand, protein immobilization can also lead to a decreased enzymatic activity due to poorer substrate accessibility [39,40]. Different strategies for protein immobilization on a surface have been described, e.g., the use of hydrogels, biopolymers or cross-linking [41].

Another method exploits the self-assembling property of hydrophobins. Hydrophobins are small, cysteine rich, amphiphilic proteins derived from fungi [42,43]. They are responsible for the water repellent surface of fungi and well-known for their ability to self-assemble at hydrophobic-hydrophilic interfaces [44,45]. Hydrophobins are divided into two classes based on their hydropathy patterns [44]. Class I hydrophobins form very stable, rodlet shaped monolayers at interfaces that can only be dissociated with strong acids [46], whereas class II aggregates can be dissolved much easier and do not form rodlet shaped aggregates [47,48]. By exploiting their ability to self-assemble, hydrophobins have already successfully been used to immobilize proteins and peptides on a surface [49,50]. Hydrophobin-mediated surface functionalization allows for the development of a stable and highly ordered surface presenting a target molecule.

For this work, we have chosen the hydrophobin Ccg2 from *Neurospora* (*N.*) *crassa* also known as EAS [51]. Ccg2 is a class I hydrophobin that forms a robust amphipathic rodlet layer on interfaces [52,53]. It contains eight cysteine residues, typical for hydrophobins that form four disulfide bridges [51,54]. The 3D-structure of Ccg2, first described by Kwan et al. [54], revealed that the protein consists of a well ordered β-barrel core, while the other protein regions appeared quite unstructured. Surface-exposed amino acids are well-separated in charged and uncharged areas, presenting a clear segregation of hydrophobic and hydrophilic regions.

By utilizing a hydrophobin functionalized surface presenting EPSPS, this work provides a proof of principle for the fast and simple detection of glyphosate in the nanomolar range. It is based on the specific inhibition of enzymatic EPSPS activity by glyphosate, which leads to a decrease in formed Pi, which is measured with the malachite green assay. Furthermore, we present a simple strategy to increase the robustness of the assay by elimination of unspecific Pi in the analyte solution.

## 2. Materials and Methods

### 2.1. Chemicals

Chemicals used were purchased from VWR International (Radnor, PA, USA) or Merck KGaA (Darmstadt, Germany). The substrates for the EPSPS enzymatic reaction, shikimate-3-phosphate trisodium salt (S3P) and phosphoenolpyruvate-monopotassium salt (PEP) as well as the pesticides chlorpyrifos, glufosinate and glyphosate´s primary degradation product aminomethyl-phosphonic acid (AMPA) were purchased from Merck KGaA. Glyphosate was obtained from Molekula (Darlington, UK).

### 2.2. Molecular Cloning of Ccg2 and Ccg2_GS_EcEPSPS

Cloning of constructs was done using standard cloning techniques including PCR, restriction and ligation. The open reading frame (ORF) of Ccg2 was synthesized without its signal peptide. The ORF of *aroA* coding for *Escherichia (E.) coli* EPSPS (EcEPSPS, gene ID: 945528, UniProt: P0A6D3) was PCR-amplified from *E. coli* DH10α (New England Biolabs GmbH, Frankfurt am Main, Germany). To create the fusion gene Ccg2_GS_EcEPSPS a glycine-serine linker sequence (G_4_S)_3_ was adhered to the 5′-region of the EcEPSPS using a modified primer. Cloning was done using *E. coli* Top10F’ (Merck KGaA) and the desired PCR products were integrated via restriction and ligation into the pET28b vector (Merck KGaA) 3’ to the (His)_6_-tag sequence, resulting in constructs pET28b_Ccg2 and pET28b_Ccg2_GS_EcEPSPS.

### 2.3. Protein Expression and Purification

The plasmids pET28b_Ccg2 and pET28b_Ccg2_GS_EcEPSPS were transformed into the expression strain *E. coli* SHuffle^®^ T7 express lysY (New England Biolabs). Transformed bacteria were grown in LB_MOPS_ medium (5 g/L yeast extract, 10 g/L peptone, 5 g/L sodium chloride, 10.5 g/L 3-(*N*-morpholino)propanesulfonic acid (MOPS), pH 7.4) with 60 mg/mL kanamycin as selective antibiotic. Protein expression was induced by adding 1 mM isopropyl-β-D-thiogalactoside (IPTG) to the culture medium at an OD_600_ of >0.4. Cultures were grown for 4 h at 30 °C, 180 rpm, harvested by centrifugation (15,000× *g*, 10 min, 4 °C) and washed twice with Tris/HCl (pH 7.5). Cells were disrupted by incubation with lysozyme, DNase, RNase and benzonase for 30 min at 37 °C under shaking followed by ultrasonic treatment.

The fusion protein Ccg2_GS_EcEPSPS, found in the soluble fraction, was purified under native conditions via the N-terminal (His)_6_-tag by Ni^2+^-affinity chromatography according to the manufacturer’s instructions (His-Bind^®^ resin, Merck KGaA). The eluted protein fractions were dialyzed twice against 1.5 L dialysis buffer (10 mM MOPS, 0.5 mM etyhlendiaminetetraacetic acid (EDTA), 5% (*v*/*v*) 99% glycerin, 1 mM dithiothreitol (DTT), [55]) for 12 h in a dialysis cassette (Slide-A-Lyzer^®^, 10,000 MWCO, Thermo Fisher Scientific, Waltham, MA, USA).

The hydrophobin Ccg2 was mainly detected in the insoluble fraction and purified as described [50,54]. Briefly, the insoluble pellet was incubated three times on a rotating wheel for 30 min in lysis buffer (8 M urea, 50 mM potassium phosphate, 50 mM sodium phosphate, 10 mM Tris, pH 8.0). Solubilized proteins were purified using the N-terminal (His)_6_-tag by Ni^2+^-affinity chromatography (His-Bind^®^ resin, Merck KGaA) according to the manufacturer’s instructions for denaturating purification. Subsequently, the proteins were concentrated by ultrafiltration using a Vivaspin20 column (5000 MWCO, Sartorius AG, Göttingen, Germany) and finally dialyzed twice for 24 h against 2 L redox-refolding dialysis buffer (10 mM glutathione reduced, 1 mM glutathione oxidized, pH 5.4, [54]). Protein concentration was determined using the Bradford method [56]. Bovine serum albumin (BSA) was used as the standard protein. Protein purification was examined by running a tricine-SDS-PAGE [57] or glycine-SDS-PAGE [58] for hydrophobins and Ccg2_GS_EcESPSP, respectively. Proteins were blotted after electrophoresis on a polyvinylidene fluoride (PVDF) membrane and detected with 6xHis monoclonal antibody (TaKaRa Bio Inc., Kusatsu, Japan) via immunodetection or stained with colloidal Coomassie brilliant blue.

### 2.4. Surface Functionalization and Contact Angle Measurement

For surface functionalization of 96-well plates (non-treated polystyrene or glass), protein solution was pipetted on the surface and incubated for 30 min at room temperature. Next, the protein solution was removed, followed by a further incubation step for 5 min. The functionalized surface was washed at least 10 times with dialysis buffer.

For water contact angle measurements, polystyrene or glass surfaces were cleaned with pure ethanol and distilled water. Surfaces were air-dried and protein solution was carefully pipetted on the surface and incubated overnight. On the next day, surfaces were washed thoroughly with distilled water and air-dried. The contact angle of a 2 µL distilled water drop was determined using the OCA20 device (Dataphysics, Filderstadt, Germany). Measurements were performed for at least 7 drops.

### 2.5. Measurement of Enzyme Activity in Solution

To determine whether the heterologous expressed enzyme derivative is active, activity measurement using the EnzCheck^®^ phosphate assay kit (Thermo Fisher Scientific, Waltham, MA, USA) was performed. The assay was carried out with 0.5 µg of purified fusion protein (Ccg2_GS_EcEPSPS) according to the manufacturer´s instructions. Substrate concentrations of 100 µM S3P and 80 µM PEP were used.

### 2.6. Measurement of Enzyme Activity of the Immobilized Proteins

Surfaces of 96-well plates were functionalized as described above (see Section 2.4). Enzyme activity was determined with the malachite green assay according to Baykov et al. [36]. After washing of the surface, glyphosate (or another analyte) in appropriate concentrations and substrates were added to final concentrations of 100 µM S3P and 20 µM PEP and filled up to a final volume of 160 µL with dialysis buffer. The reaction was stopped after a 45 min incubation at room temperature by adding 40 µL of malachite green working solution (5 mL malachite green solution (125 mL ddH_2_O, 25 mL sulfuric acid (concentrated), 183 mg malachite green), 1.25 mL 7.5% ammonium molybdate solution, 0.1 mL 11% Tween20). Malachite green working solution was incubated for 20 min in the dark (RT) and absorbance was measured using a microplate reader device (Infinite 200, Tecan Group Ltd., Männedorf, Switzerland) at a wavelength of 630 nm. Unless otherwise stated, assays were performed in triplicates and repeated independently 3 times. Error bars show standard deviations. A blank value with all reagents in the absence of a protein-coated surface was subtracted from each sample as background control.

### 2.7. Statistics

Data were analyzed for significance using a one-sided student’s T-Test using Microsoft Excel 2010.

## 3. Results

### 3.1. Cloning of Fusion Genes and Protein Purification

**Figure 1:**
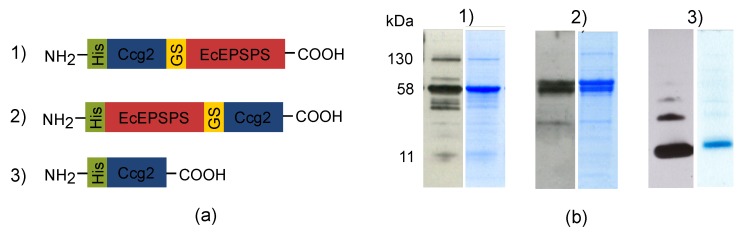
Utilized proteins for this study. (**a**) Schematic representation of Ccg2 and Ccg2-EPSPS fusion proteins; (**b**) Western blots (left lanes) and Coomassie stained gel strips (right lanes) after expression and purification of the proteins. For Western blot analysis the samples were electrophoretically separated, subsequently transferred to a PVDF membrane and probed with 6xHis monoclonal-antibody. Molecular masses (kDa) are indicated on the left. The molecular masses of the fusion proteins (1 + 2) and Ccg2 (3) are ca. 58 kDa and 11 kDa, respectively.

To prepare a functionalized surface presenting the *E. coli* enzyme 5-enolpyruvyl-shikimate-3-phosphate synthase (EcEPSPS) to detect glyphosate, we investigated two different variants of fusion proteins between the hydrophobin Ccg2 and the enzyme EcEPSPS, both separated by a flexible glycine-serine-linker (G_4_S)_3_. This strategy was used to figure out which fusion protein better meets the criteria for our approach. One of the constructs carries the hydrophobin Ccg2 at the N-terminal and the EcEPSPS at the C-terminal end of the linker, while the other construct was designed vice versa (see Figure 1a). Both constructs were cloned into the pET28b vector, providing a N-terminal (His)_6_-tag, expressed in *E. coli* SHuffle^®^ T7 express lysY and purified by native Ni^2+^-affinity purification. Furthermore, we expressed the hydrophobin Ccg2 lacking a fusion partner. The SHuffle^®^ T7 express lysY strain was chosen due to its disulfide isomerase activity, which should support the formation of disulfide-bridges in the cytoplasm to keep the hydrophobins in a soluble form [59,60]. Nevertheless, the hydrophobins were almost exclusively found in the pellet fraction and had to be purified via denaturating purification. By contrast, the fusion proteins were found in the soluble fraction and thus purified via native Ni^2+^-affinity purification.

In Figure 1b the results of the Western blot analysis with 6xHis monoclonal-antibody and Coomassie staining of the electrophoretically separated proteins are shown. Expression and purification of the different proteins were successful. The fusion protein Ccg2_GS_EcEPSPS is visible as a dominant band at a molecular mass of ca. 58 kDa (Figure 1b). In case of the EcEPSPS_GS_Ccg2 fusion protein, two neighboring bands of around 58 kDa are seen, which may reflect isomeric forms resulting from incomplete reduction of disulfide bridges prior to SDS-PAGE [61]. In case of Ccg2, a strong signal at ca. 11 kDa indicates the successful expression and purification of the hydrophobin. Protein bands with a lower molecular mass might reflect degradation products of the fusion proteins, while signals at higher molecular masses probably are due to protein multimerization [50].

The purified proteins were applied for surface functionalization.

### 3.2. Self-Assembling Properties and Activity Measurement in Solution

After purification the proteins were tested for their functionality, i.e., the ability for self-assembly due to the hydrophobin and for enzymatic activity due to the EcEPSPS. Preliminary tests revealed a higher enzymatic activity of the fusion protein with the N-terminal Ccg2 (Figure 1a, construct 1, see Figure S1). Only this fusion protein was used for further experiments.

Self-assembly properties were tested using water contact angle measurement. The amphiphilic hydrophobins self-assemble at interfaces and change surface properties regarding their wettability with water. In the case of successful hydrophobin-layer formation a hydrophilic glass surface will turn hydrophobic, whereas a hydrophobic polystyrene surface will become hydrophilic. Respective surfaces were functionalized as described (see Section 2.4) and subjected to water contact angle measurements.

**Figure 2:**
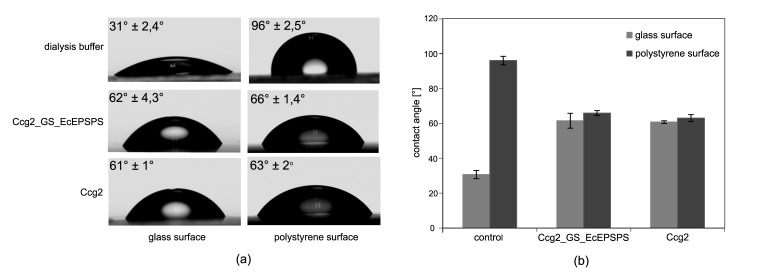
Water contact angle measurements on glass and polystyrene surfaces. The respective surface was treated with protein solution and then washed thoroughly with distilled water. Contact angle of a 2 µL water droplet was determined using the OCA20 (Dataphysics) contact angle measuring instrument. (**a**) Representative drop shapes of surfaces functionalized with dialysis buffer (control), fusion protein (Ccg2_GS_EcEPSPS)-and hydrophobin (Ccg2)-solution. Mean values of measured contact angles are depicted in the upper left corner. (**b**) Bar graph of measured contact angles with standard deviation for 7 different drops (except Ccg2_GS_EcEPSPS (glass) = 6 drops).

Figure 2 shows the results of the contact angle measurements on glass and polystyrene surfaces for the immobilization of fusion protein Ccg2_GS_EcEPSPS and the hydrophobin Ccg2. As a control, a surface treated with dialysis buffer was used.

The glass surface shows a contact angle after dialysis buffer treatment of 31° ± 2.4°, which is defined as hydrophilic [62]. Glass surfaces incubated with Ccg2_GS_EcEPSPS or Ccg2 are more hydrophobic with a contact angle of ~61° indicating a successful functionalization.

On a polystyrene surface, dialysis buffer shows a contact angle of 96° ± 2.5° which is commonly denoted as hydrophobic [62]. Upon functionalization the polystyrene surface became more hydrophilic: With the fusion protein and hydrophobin, the contact angle decreases to 66° and to 63°, respectively.

The results of the contact angle measurement indicate the self-assembly properties of both, the purified hydrophobin and the fusion protein. This is in line with former reports showing that the self-assembling properties of hydrophobins are not influenced by modification of their N- or C-termini [49,63,64,65]. However, contact angle measurements only hint at a successful functionalization via hydrophobin self-assembly because proteins can also change the wettability of surfaces with water in an unspecific manner, e.g., through binding mediated by hydrophobic patches. In contrast, self-assembled hydrophobin monolayers are very stable and can only be removed with strong acids [47,66,67,68]. Nevertheless, our results indicate that both the purified hydrophobin and the hydrophobin-EcEPSPS fusion protein self-assemble at interfaces and can be used to functionalize glass and polystyrene surfaces.

**Figure 3:**
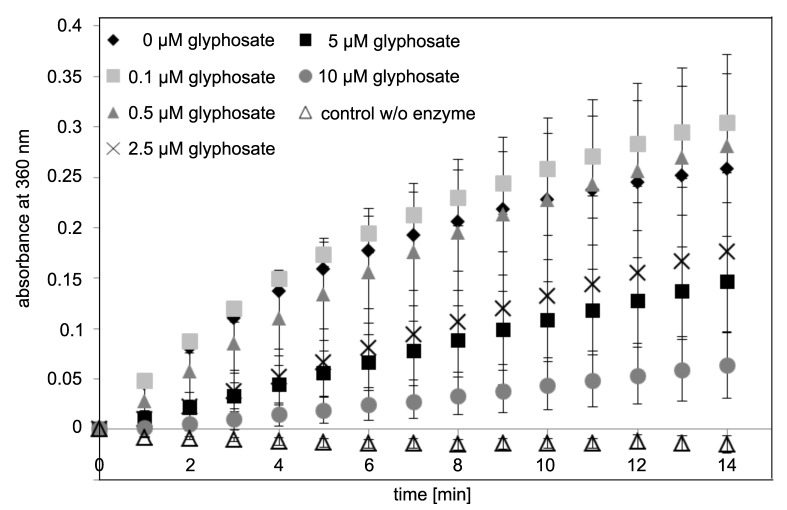
Activity measurement of Ccg2_GS_EcEPSPS (0.05 µM) in solution by detecting the formation of inorganic phosphate (Pi) using the EnzCheck^TM^ Phosphate Assay Kit using photometry at a wavelength of 360 nm. Different glyphosate concentrations were added to the reaction, and the activity was measured over 14 min. Standard deviations derived from mean values of 2 independent measurements.

Next, we tested the enzymatic activity of Ccg2_GS_EcEPSPS in solution. Reaction of EPSPS with its substrates S3P and PEP leads to the formation of Pi, which can be measured by photometry at a wavelength of 360 nm using the EnzCheck^TM^ Phosphate Assay kit. The formation of Pi for 14 min was determined with different glyphosate concentrations for 0.05 µM fusion protein in solution (Figure 3).

After one minute, the absorbance at a wavelength of 360 nm increased due to the enzymatic activity. Already a concentration of 2.5 µM glyphosate leads to a decrease in enzymatic activity compared to the control without glyphosate. The results suggest that the fusion protein Ccg2_GS_EcEPSPS exhibits an enzymatic activity that is inhibited by glyphosate, which was a prerequisite for the aimed assay.

### 3.3. Surface Functionalization and Determination of Occupancy Ratio

The malachite green assay, an alternative method for the detection of Pi, is cheaper than the EnzCheck^TM^ Phosphate Assay kit and proved to be more suitable for activity measurement of functionalized surfaces in initial experiments (data not shown). Functionalization of 96-well plates and the malachite green assay were performed as described (see Section 2.4 and Section 2.6). As the molecular mass of the enzyme is about 4 times larger than that of the hydrophobin, it is possible that steric hindrance interferes with the fusion protein’s ability to self-assemble. Therefore, we tested different molar ratios of fusion protein to hydrophobin to find an appropriate ratio for the surface functionalization. The necessity for an optimization of respective ratios has recently been reported for sensor applications [50].

**Figure 4:**
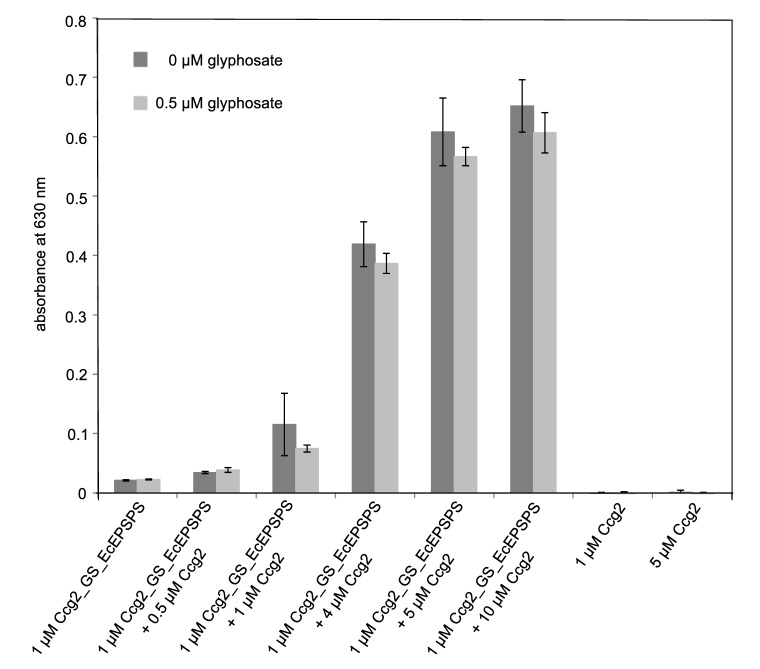
Activity measurements using the malachite green assay with polystyrene surfaces functionalized with Ccg2_GS_EcEPSPS and Ccg2 in different ratios (triplicate measurement). Absorbance at a wavelength of 630 nm was determined using photometry.

Figure 4 shows the result of the malachite green assay with polystyrene surfaces functionalized with different ratios of Ccg2_GS_EcEPSPS to Ccg2. Functionalization of polystyrene surfaces with only 1 µM of the fusion protein leads to a low enzymatic activity, and inhibition by 0.5 µM glyphosate cannot be detected. Interestingly, raising the concentration of Ccg2 from 0 µM to 10 µM at constant fusion protein concentration results in an increase in the enzymatic activity. Beginning at a 1:1 ratio of hydrophobin to fusion protein, an inhibition of enzymatic activity by 0.5 µM glyphosate is detectable. Functionalization of the surface with hydrophobin yields no detectable signal at 630 nm, documenting that the measured absorbance is due to EcEPSPS activity on the surface. For the further experiments, we chose a ratio of 1 µM Ccg2_GS_EcEPSPS to 5 µM Ccg2, combining appropriate self-assembly property with the possibility for detection of the inhibition of the enzymatic activity by 0.5 µM glyphosate.

### 3.4. Concentration-Dependent Inhibition of EPSPS Enzymatic Activity by Glyphosate

Next, we tested functionalized surfaces for the detection of different glyphosate concentrations ranging from 0 to 1 µM and aimed at optimization of the assay. For this purpose, we used a 96-well plate with glass bottom and a reaction buffer with pH 10. The rationale is that glyphosate is deprotonated at basic pH values, and inhibition of EPSPS activity is more efficient in this form [69,70].

In order to show that the assay is not restricted to polystyrene, a glass plate was used herein. Regarding future applications, this information is important as different materials can be used to prepare ready-to-use chips. The results on polystyrene surface were similar but with slightly higher standard deviations (see Figure S2). Thus both materials are appropriate to generate robust results with the self-assembled layers.

**Figure 5:**
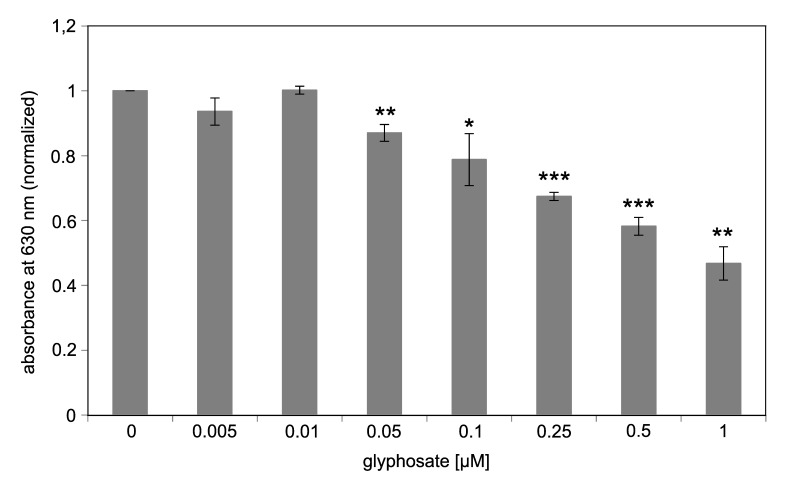
Inhibition of immobilized Ccg2_GS_EcEPSPS by glyphosate. Measurement of EcEPSPS activity using the malachite green assay with a glass surface functionalized with 1 µM Ccg2_GS_EcEPSPS:5 µM Ccg2. Results were normalized to the sample without glyphosate (0 µM). The detection limit is 50 nM glyphosate. *** *p* ≤ 0.001; ** *p* ≤ 0.01; * *p* ≤ 0.05. Absorbance at a wavelength of 630 nm was determined using photometry.

Figure 5 shows the inhibition of immobilized EcEPSPS with different glyphosate concentrations. Results were normalized to the sample without glyphosate (0 µM). Low glyphosate concentrations of 0.005 and 0.01 µM cannot be detected, as impairment of the enzymatic activity by glyphosate cannot be seen. However, starting from 0.05 µM glyphosate a nearly linear decrease in the absorption at 630 nm compared to the control without glyphosate can be observed. These data document that a concentration-dependent inhibition of the enzymatic activity by glyphosate and in turn detection of the pesticide by the functionalized surfaces and the malachite green assay is feasible.

### 3.5. Cross Reactivity of the Assay

We next investigated the specificity of the assay. Glyphosate is, amongst many others, an organophosphonate pesticide. To determine cross reactivity, another organophosphonate pesticide, glufosinate, an organophosphate, chlorpyrifos, and the first main degradation product of glyphosate, AMPA, were tested.

**Figure 6:**
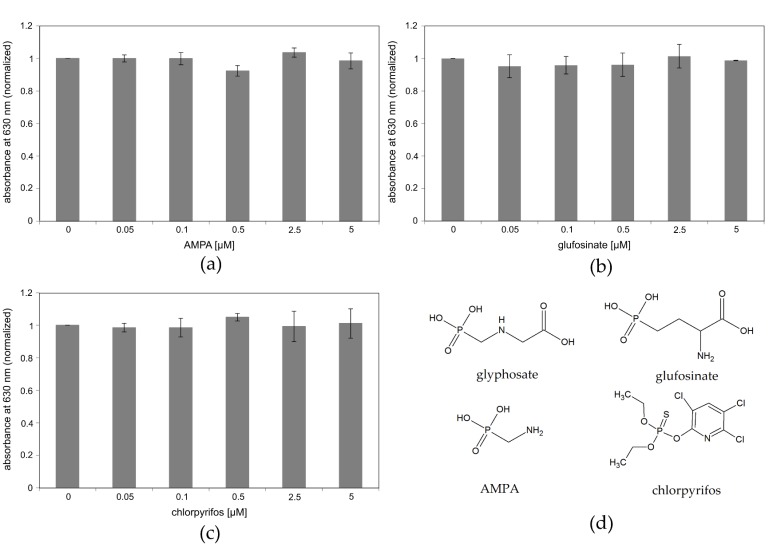
Measurement of cross-reactivity of the functionalized surface (1 µM Ccg2_GS_EcEPSPS:5 µM Ccg2, polystyrene) with the malachite green assay towards other pesticides. Results were normalized to the sample without glyphosate (0 µM). Absorbance at a wavelength of 630 nm was determined using photometry. (**a**) Cross-reactivity for AMPA (aminomethylphosphonic acid), the primary degradation product of glyphosate. (**b**) Cross-reactivity for glufosinate, a member of amino acid-derived organophosphonate pesticides. (**c**) Cross-reactivity for the insecticide chlorpyrifos, belonging to the group of organophosphate pesticides. (**d**) Chemical structure of glyphosate and the three tested chemicals.

None of the three tested substances showed any influence on the enzymatic activity of immobilized EcEPSPS in a concentration range between 0 µM and 5 µM (Figure 6a–c). This holds even true for glufosinate, which is structurally quite similar to glyphosate (Figure 6d). Given that, to our knowledge, no other pesticide targets the EPSPS, the assay provides a highly specific detection system for glyphosate. However, the robustness of the assay to parameters like salt and metal ions has still to be determined.

### 3.6. Pre-Incubation of the Functionalized Surface with Glyphosate Solution

As the assay is based on the detection of Pi it is important that the analyte solution is free of phosphate. When testing the removal of phosphate with different phosphate-binding substances like magnetite or calcium peroxide, we noticed that this treatment also partially removed glyphosate out of solutions (data not shown). To circumvent this problem, we used a different approach.

Enzymatic reactions are equilibrium reactions. It is known that glyphosate inhibits the EPSPS in a slowly reversible manner [32]. Commonly, it is supposed that binding of glyphosate to the open form of EPSPS mainly occurs after binding of S3P [71]. Nevertheless, we pre-incubated the functionalized surfaces with a glyphosate containing solution, removed it carefully after 15 min incubation and measured the enzymatic activity with the malachite green assay. To test whether phosphate residues in the analyte solution will influence the results, we spiked the glyphosate solution with different concentrations of potassium phosphate.

**Figure 7:**
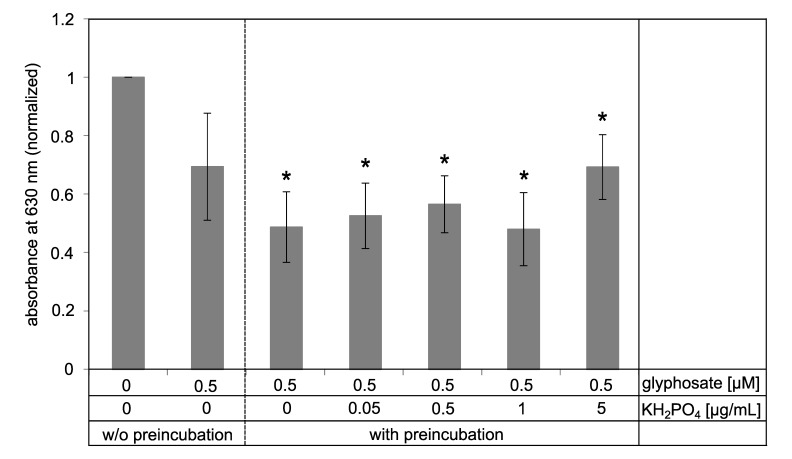
Malachite green assay with preincubation of the surface for 15 min with a solution containing 0.5 µM glyphosate and different phosphate concentrations. After removal of preincubation solution, the assay was performed as described. Samples with 0 and 0.5 µM glyphosate without preincubation were used as positive controls. Results were normalized to the sample without glyphosate (0 µM). Absorbance at a wavelength of 630 nm was determined using photometry. * *p*-value ≤ 0.05 compared to sample 0 µM glyphosate w/o preincubation.

Pre-incubation with 0.5 µM glyphosate led to a significantly reduced absorbance at 630 nm compared to the control without glyphosate (Figure 7). Interestingly, increasing phosphate concentrations in the pre-incubation solution had only a slight effect on the assay. Thus, the complete removal of phosphate from the analyte solution appears not to be essential for the pre-incubation method.

**Figure 8:**
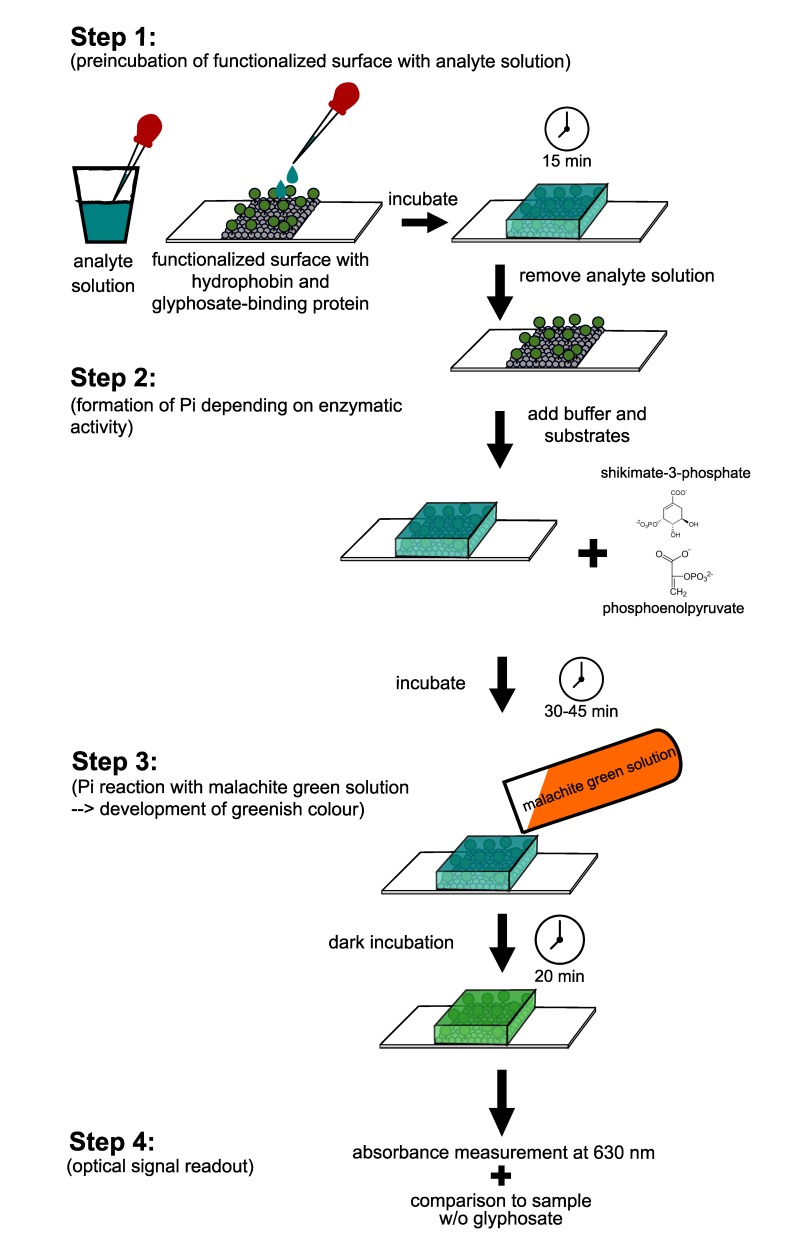
Workflow scheme of the glyphosate detection assay. For details see text.

Based on these findings, we developed a workflow for the proposed assay based on the functionalized surfaces and the malachite green assay as a fast and simple detection system for glyphosate (Figure 8).

In that, our assay consists of 4 steps. In the first step the “ready-to-use” functionalized surface is pre-incubated with the analyte solution for 1 min. After careful removal of the analyte solution, the buffer and the two substrates necessary for the enzymatic activity, PEP and S3P, respectively, are added in step 2, followed by incubation for 30 to 45 min at room temperature. In step 3 the reaction is stopped by adding the malachite green working solution, followed by incubation for 10 to 20 min at room temperature, preferably in the dark. Step 4 comprises the signal read out using a photometer or a spectrophotometer. Absorbance can be measured between 620 and 660 nm [33,36] and has to be compared to a control sample without glyphosate.

The ready-to-use surface allows for a fast and easy assay. It takes about 60 min and requires only a pipette with tips, the reagents and a photometer. By using a hand-held photometer, the assay will also be applicable for in-place measurements in the field.

## 4. Discussion

The aim of our study was to provide an assay for the detection of glyphosate, which is easy to handle, fast and needs affordable equipment. To this end, we functionalized surfaces with a fusion protein consisting of the Ccg2 hydrophobin and EcEPSPS as the specific pesticide target protein [31]. Impairment of the enzymatic function leads to a decreased production of Pi, which is detected via a malachite green assay.

### 4.1. Contact Angle Measurements

Using contact angle measurements, we were able to show that the purified recombinant proteins keep their ability for self-assembly. Functionalization of either glass or polystyrene surfaces with the fusion protein or hydrophobin leads to the expected change in the water contact angle compared to the corresponding control. Water contact angles were reduced by about 30° on hydrophobic polystyrene and increased by about 30° on hydrophilic glass. This is in line with previously reported findings [50,72].

### 4.2. Activity Measurement and Glyphosate Inhibition

The fusion of two proteins can lead to functional loss, e.g., if one partner protein covers the active center of the other protein. To determine whether the recombinant Ccg2_GS_EcEPSPS fusion protein is enzymatically active, we measured its activity in solution by detection of Pi generated by the enzyme. We used the EnzCheck^TM^ Phosphate Assay kit to record the enzyme kinetics. As can be seen in Figure 3, the enzyme shows the typical substrate-saturation curve of an enzymatic reaction, proving that the enzymatic function of EcEPSPS is not markedly affected by the fusion to the hydrophobin. Inhibition by glyphosate can be detected at concentrations exceeding 2.5 µM of the pesticide.

Immobilization of an enzyme in an active manner requires correct orientation of the protein on the surface, so that the active center is accessible for substrate binding. To avoid steric hindrance between enzyme molecules at the surface, we tested different molar ratios of fusion proteins to hydrophobins. Mixing of the fusion protein with hydrophobin for surface functionalization leads to an increased signal to noise ratio and allows detecting the inhibition by glyphosate. Fokina et al. [65] recently tested different molar ratios of a DewA-LaccaseC fusion protein to pure DewA hydrophobin. In contrast to our results the best laccase activity was obtained by coating the surface only with fusion proteins. In line with our results Takatsuji et al. [64] reported that a 1:1 to 1:19 mixture of a glucose oxidase-hydrophobin fusion protein and the respective hydrophobin HFBI exhibits the highest enzymatic activity. We found that a mixture of 1 µM Ccg2_GS_EcEPSPS to 5 µM Ccg2 is a suitable ratio to perform the surface associated malachite green assay, but other ratios might be applicable as well.

Using different glyphosate concentrations we could show that immobilization of the EcEPSPS via fusion with a hydrophobin is advantageous and, under the tested conditions, leads to a 10 times lower detection limit for glyphosate compared to measurement in solution (Figure 3 and Figure 5). Our assay exhibits a detection limit of 50 nM (8.45 ng/mL) glyphosate. ELISA tests for glyphosate gain a detection limit of 0.6 ng/mL [17], which is around 15 times more sensitive than our assay. Nevertheless, glyphosate concentrations reported in beer and wine are in the range of our detection limit [8,73]. Furthermore, it is possible to concentrate the analyte solutions as it was done for ELISA [74].

We used chemically related and unrelated substances to test the specificity of our assay. AMPA, the main degradation product of glyphosate, shares a highly similar molecular structure and has been reported to cross-react with glyphosate detection methods [24,74]. This may falsify data as AMPA is not only a degradation product of glyphosate but also of some detergents and other amino-polyphosphonates [75,76,77]. As expected because AMPA is no active inhibitor of EPSPS, our assay shows no cross-reactivity in the tested concentration range for AMPA. The same holds true for glufosinate, which is like glyphosate an amino-acid derived phosphonate, and the insecticide chlorpyrifos, which was included in the test as a chemically unrelated substance. These results show that our assay benefits from the use of the natural target enzyme of glyphosate. To our knowledge, no other pesticide is known that inhibits EPSPS except for an artificial S3P-derivative which was used in studies on glyphosate´s mode of action [78] and some glyphosate derivatives [79].

### 4.3. Practicability of the Assay

The proposed assay relies on the detection of phosphate from the enzymatic reaction using the malachite green assay. The use of phosphate binding substances to remove phosphate from the analyte solution prior to the enzymatic activity testing led to a partial removal of glyphosate from the solution. This removal can occur due to the chelating properties of glyphosate [80,81] as metal salts were used as phosphate binding substances. In an alternative approach, we removed the analyte solution from the EPSPS immobilized surface after 15 min thoroughly before performing the malachite green assay. This approach allowed to monitor the glyphosate-dependent enzyme inhibition, even in the presence of phosphate-spiked glyphosate solution. While phosphate concentrations up to 1 µg/mL did not disturb the assay, a slight increase in absorption was observed for higher phosphate concentrations (5 µg/mL).

It has to be noted that pre-incubation of the chip surface with analyte solutions containing glyphosate provided reliable results. This finding is interesting as it is commonly accepted that binding of glyphosate to EPSPS is dependent on the presence of an S3P∙enzyme complex [32,71,82]. We argue that glyphosate interaction with EPSPS occurs already strong enough in the open enzyme form, resulting in an enzymatic inhibition even after removal of analyte solution during the timeframe of our developed assay. A previous report supports such a hypothesis as glyphosate binding to EPSPS without S3P was reported with lower affinity [83]. However, using our pre-incubation approach, the robustness of the assay has to be further investigated.

Moreover, detailed experiments also have to be done to test other robustness parameters of the proposed assay including salt and metal ion content, as well as pH of the analyte.

## 5. Conclusions

In this paper we describe a fast and easy detection assay for glyphosate by taking advantage of surfaces functionalized with a hydrophobin-EcEPSPS fusion protein together with the malachite green assay to determine inorganic phosphate concentrations. The assay shows excellent selectivity comparable or even superior to known ELISA tests. The assay´s detection limit is higher than that of commercially available ELISA kits, but it is very easy to handle and to apply. Notwithstanding this aspect and tests of robustness at different solution parameters, the strengths of the described assay are the short assay time, its easy workability and the affordable lab equipment needed.

## 6. Patents

This work is part of a patent application “Verfahren zur Detektion von Analyten auf Basis immobilisierter Proteine” (application number: DE 10 2018 130 133.2).

## Supplementary Materials

The following are available online at https://www.mdpi.com/2079-6374/9/3/104/s1, Figure S1: Activity measurement for different proteins after immobilization, Figure S2: Inhibition of immobilized Ccg2_GS_EcEPSPS by glyphosate on polystyrene.
