# Supplementary material for: Surface Functionalization by Hydrophobin-EPSPS Fusion Protein Allows for the Fast and Simple Detection of Glyphosate
Source: Biosensors (Basel). 2019 Aug 29;9(3):104. doi: 10.3390/bios9030104 (PMC6784374; doi:10.3390/bios9030104)
Supplement: Supplementary file 1 [file biosensors-09-00104-s001.pdf]

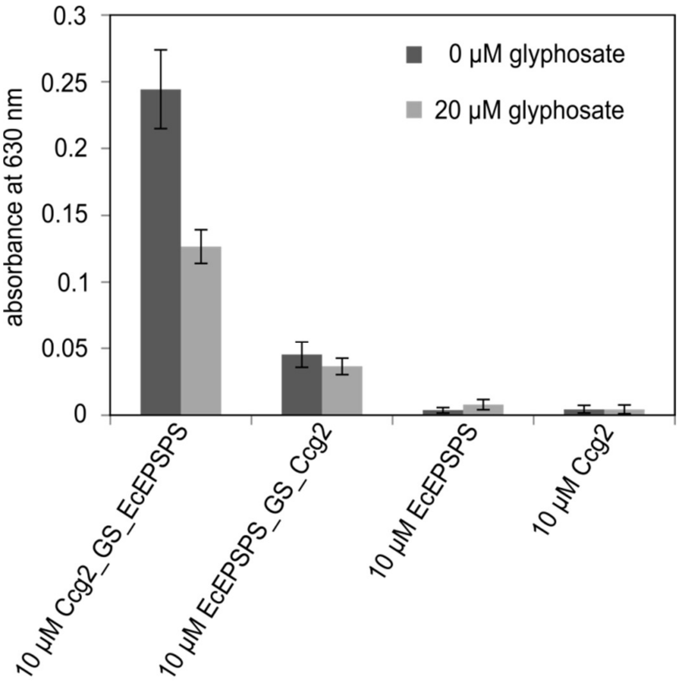

Figure S1. Activity measurement for different proteins after immobilization. Proteinsolutions were incubated for 15 min in a polystyrene 96-well plate. Malachite green assay was performed as described in materials and methods (see Section 2.6). Substrate concentrations were 100 and 80 µM for S3P and PEP, respectively.

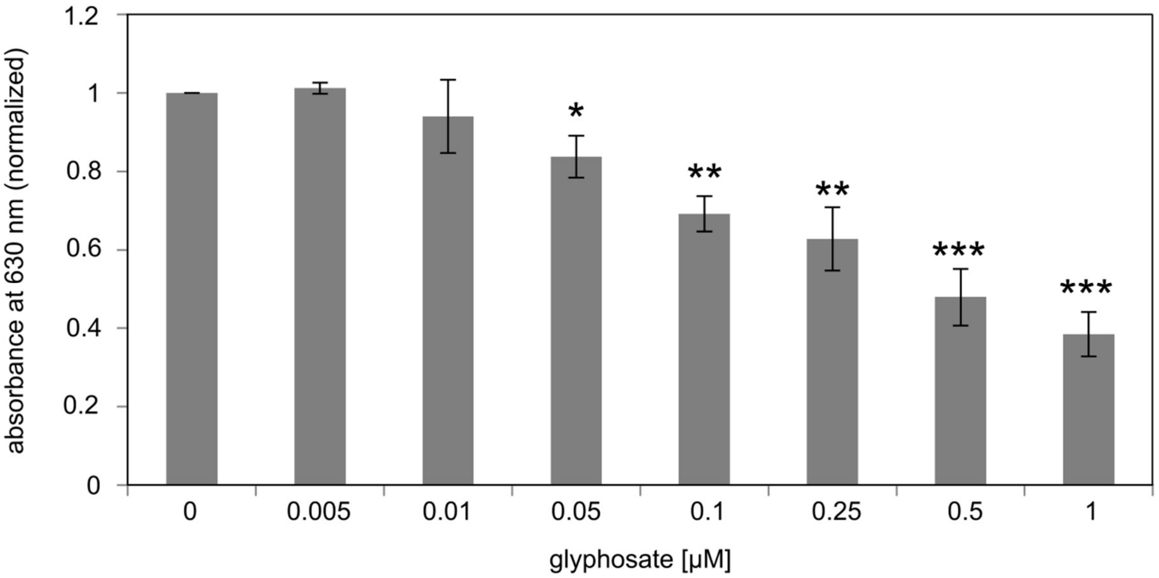

Figure S2. Inhibition of immobilized Ccg2_GS_EcEPSPS by glyphosate. Measurement of EcEPSPS activity using the malachite green assay with a polystyrene surface functionalized with 1 µM Ccg2_GS_EcEPSPS:5 µM Ccg2. Results were normalized to the sample without glyphosate (0 µM). The detection limit is 50 nM glyphosate. *** $p \leq 0.001$; ** $p \leq 0.01$; * $p \leq 0.05$. Absorbance at a wavelength of 630 nm was determined using photometry.
